# Therapeutics Development for Alagille Syndrome

**DOI:** 10.3389/fphar.2021.704586

**Published:** 2021-08-23

**Authors:** Phillip Sanchez, Atena Farkhondeh, Ivan Pavlinov, Karsten Baumgaertel, Steven Rodems, Wei Zheng

**Affiliations:** ^1^ National Center for Advancing Translational Sciences, National Institutes of Health, Bethesda, MD, United States; ^2^ Travere Therapeutics, San Diego, CA, United States

**Keywords:** alagille syndrome, JAG1, notch signaling pathway, liver, bile duct, drug development

## Abstract

Advancements in treatment for the rare genetic disorder known as Alagille Syndrome (ALGS) have been regrettably slow. The large variety of mutations to the JAG1 and NOTCH2 genes which lead to ALGS pose a unique challenge for developing targeted treatments. Due to the central role of the Notch signaling pathway in several cancers, traditional treatment modalities which compensate for the loss in activity caused by mutation are rightly excluded. Unfortunately, current treatment plans for ALGS focus on relieving symptoms of the disorder and do not address the underlying causes of disease. Here we review several of the current and potential key technologies and strategies which may yield a significant leap in developing targeted therapies for this disorder.

## Introduction

In 1975, a set of children suffering from cholestatic disease were identified to share several symptomatic similarities, distinct from others with comparable biliary dysfunctions. Characteristic facies as well as renal, vertebral and cardiac abnormalities allowed Dr. Daniel Alagille to append a single etiology, later named after him, to these children (Alagille et al., 1975). With an incidence of 1:30,000 to 1:50,000 births, the autosomal dominant Alagille Syndrome (ALGS) is a result of Notch signaling dysfunction caused by gene mutations mostly in JAG1 and NOTCH2 (Kamath et al., 2018). The most common and debilitating disease hallmark among ALGS patients is bile duct paucity, with almost all patients exhibiting cholestatic disease. Most patients experience intractable pruritis and the presence of xanthomas, localized cholesterol and fats deposits under the skin caused by this liver dysfunction. Due to the somatic nature of these mutations to Notch signaling however, several other organ systems are affected including kidney, heart, eye, nervous system, and bone. Because of the highly-variable nature of ALGS presentation, current therapeutic management is focused on addressing each patient’s symptoms individually. The lack of effective and targeted drugs for ALGS treatment therefore constitutes an unmet medical need.

JAG1 (Jagged1) is a transmembrane ligand of the Notch signaling pathway. Partial loss of JAG1 protein due to a mutation in one allele of the JAG1 gene is sufficient to disrupt proper bile duct development, resulting in bile duct paucity seen in ALGS (Hofmann et al., 2010). Recent analysis of patients with JAG1 variants has identified mutations to all 26 exons. Haploinsufficiency caused by truncation or early transcriptional termination of JAG1 account for 83% of mutations seen in ALGS patients (Gilbert et al., 2019). NOTCH2 variants are far less often designated as the sole cause of ALGS, occurring in less than 3% of patients from a recent analysis. Comparatively, mutations in NOTCH2 are more likely to be missense (68%) than in JAG1 (15%) (Gilbert et al., 2019). Although haploinsufficiency might suggest a potential for introduction of exogenous JAG1, it is not without risk. The introduction of high-levels of JAG1 can dramatically increase the risk of hyperplasia and cancer since cell-cell based contact inhibition and proliferation is greatly influenced by Notch signaling (Aster et al., 2017). Strategies focused on developing targeted therapies, which restore physiological levels of Notch signaling, will yield treatments which address underlying causes of ALGS rather than just their symptoms.

### Clinical Presentations, Treatment, and Prognosis

Alagille syndrome presents with several, often readily-observable, phenotypic traits. Distinctive facies with characteristic pointed chin, broad forehead and hypertelorism are included in diagnostic criteria. Pulmonary stenosis is frequently observed, leading to cardiac arrythmias in 63–98% of patients (Spinner et al., 1993; Kamath et al., 2018). Butterfly vertebrae are also observed, albeit less frequently and without significant symptoms. Posterior embryotoxon is the primary ophthalmological presentation for ALGS, exhibited in 78–89% of patients (Spinner et al., 1993). Renal anomalies are identified in significant (∼58.9%) subset of ALGS patients, whether or not JAG1 mutations are observed (Kamath et al., 2012).

The aforementioned characteristics are important diagnostic criteria and include bile duct paucity which is manifested in the vast majority of patients with ALGS. Symptoms of liver dysfunction include pruritis, an intractable feeling of itchy skin, caused by elevated serum bile acids as seen in other cholestatic diseases like biliary atresia. Pruritis is a major complaint of children and young patients with ALGS. Conventional treatment options for pruritus are often not effective leading to reduced quality of life for these patients. A recent survey identified 42% of parents of children with ALGS are dissatisfied with current therapeutic approaches, with many stating that attempts to reduce pruritis are wholly ineffective (Patient Family Survey, 2019).

**FIGURE 1:**
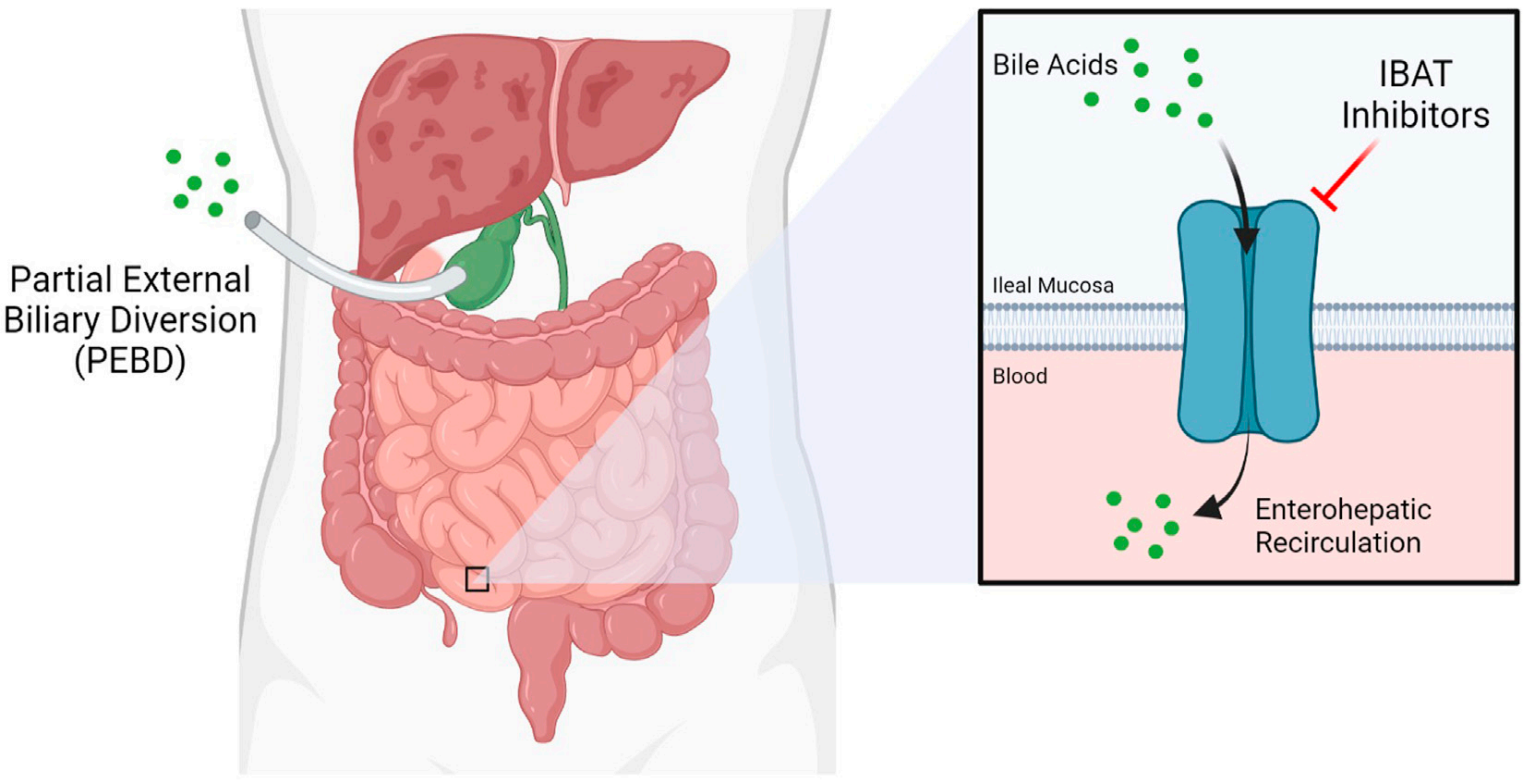
Current therapeutic approaches for Alagille Syndrome include inhibitors of the Ileal Bile Acid Transporter (IBAT) such as maralixibat, which limit enterohepatic circulation, and Partial External Biliary Diversion (PEBD), a highly invasive procedure.

Presently, the only effective treatment options for liver disease in ALGS are highly invasive. Partial external biliary diversion (PEBD) is one such approach, aiming at reducing overall bile acids present in the blood (Figure 1) (Mattei et al., 2006). PEBD often has moderate success with reducing pruritis and xanthomas, though it is not necessarily sufficient to avoid later liver transplant (Wang et al., 2017a). End-stage liver disease is treated with liver transplantation, often required in cases of neonatal ALGS which are among the direst of cases. Unfortunately, unexpected and rapid worsening of overall health in patients who need a liver transplant accounts for significant mortality in ALGS, especially within the neonate population (Lykavieris et al., 2001). Moreover, such an invasive procedure is not without risk (Kamath et al., 2018). For instance, the long-term use of immunosuppressants designed to prevent the rejection of the donor liver can trigger or aggravate issues in the kidney, often necessitating a subsequent kidney transplant (Olyaei et al., 2001). Transplant-free survival is estimated at around 24% to the age of 18.5, demonstrating the need for new treatment paradigms (Kamath et al., 2020).

### Notch Signaling and ALGS

The Notch signaling pathway is a highly conserved and indispensable component of cell-cell based signaling. Notch receptors on a signal receiving cell undergo regulated proteolysis after binding to a Notch ligand, like JAG1, from a signal transmitting cell. After several proteolytic steps on the inner membrane leaflet, the Notch intracellular domain (NICD) of the receptor is released and translocates to the nucleus. In Notch signal transduction only one signal, the NICD, is ever transmitted after proteolysis of the initial receptor. With no intermediate secondary messengers for amplification, expression of Notch target genes which are held in an “off” state by co-repressors is turned “on” by the NICD. Not surprisingly, this tight regulation of transcription strongly influences a variety of important developmental and proliferation target genes (Kopan and Ilagan, 2009).

**FIGURE 2:**
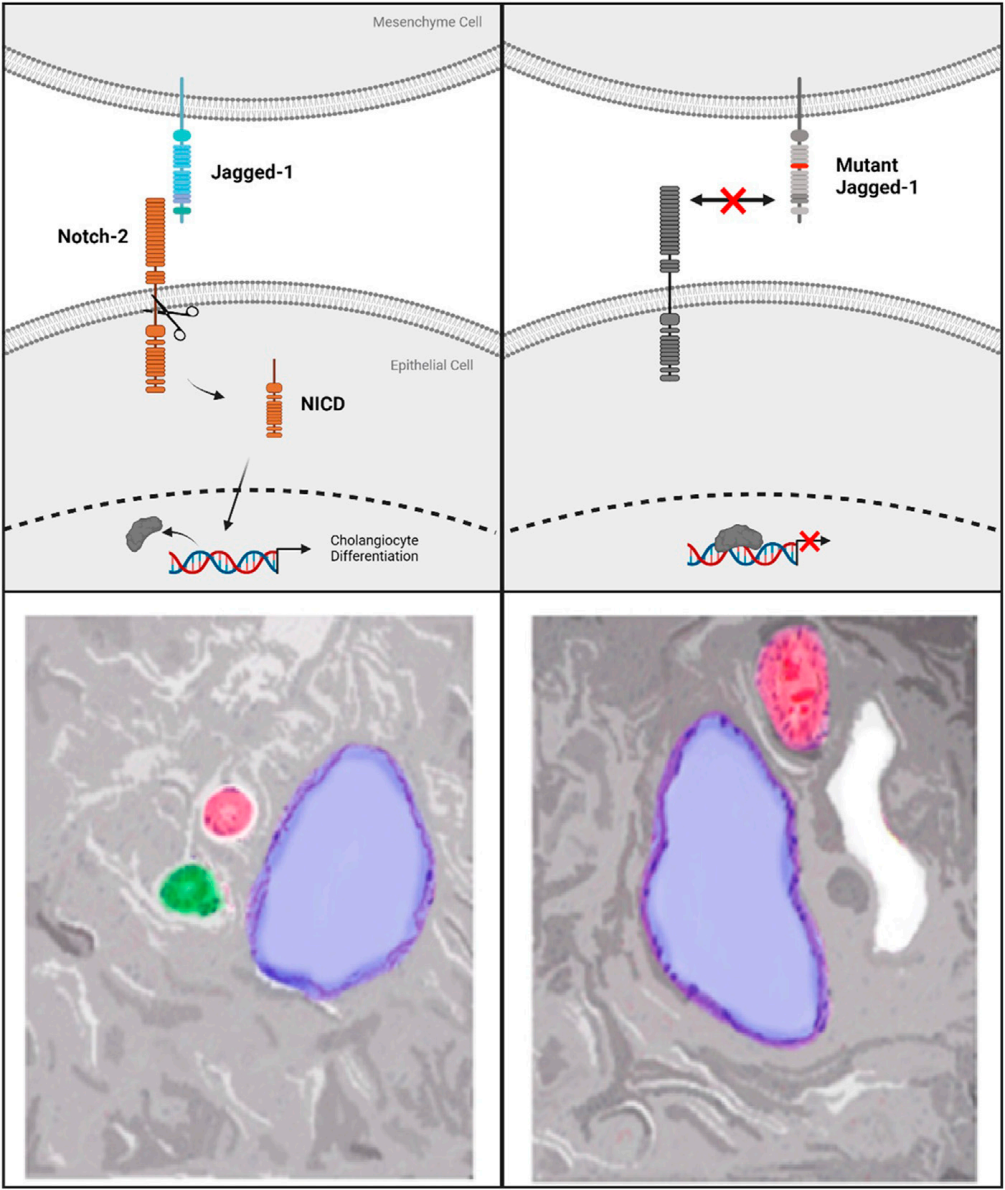
**Top left **panel shows standard Notch-signaling *via* JAG1-NOTCH2 contact causing Notch-intracellular domain (NICD) cleavage and translocation to the nucleus. **Top right** panel indicates the lack of co-repressor displacement caused by a mutation to Jagged-1 which prevents Notch-2 activation and cleavage. **Bottom Left** panel shows normal hepatic triad with Hepatic Vein (blue), Hepatic Artery (red), Bile duct (green). On the **bottom right** is an example of the lack of bile ducts observed in Alagille Syndrome caused by mutations to Notch signaling components. ALGS livers also frequently present with cysts, in white, in place of functional bile ducts.

Notch signaling is a required feature of biliary genesis during liver development with Notch2 accounting for the majority of Notch receptor expressed in fetal livers. Coordination of contact signaling between JAG1 and Notch2 expressing cells results in terminal differentiation to cholangiocytes in the Notch2 expressing hepatoblasts (Freeburg and Goessling, 2020). In histopathological analysis of ALGS livers, a distinct dearth of staining for cholangiocyte-specific signaling factors is observed (Fabris et al., 2007). Further work involving 3D-spheroid hepatocyte co-cultures indicates a critical role for the JAG1 signal specifically from portal vein mesenchyme cells (PVC). This signal instructs the formation of relevant ductal structures by biliary epithelial cells (BEC) which stain positively for the marker Cytokeratin 19 (CK-19) (Hofmann et al., 2010). Additionally, mutant JAG1 in PVCs is responsible for several instructive phenotypes; namely improper spheroid formation, disrupted luminal development and loss of CK-19 staining. Due to the loss of functional JAG1 caused by ALGS-associated mutations, cholangiocyte specification is reduced, preventing proper biliary structural development (Figure 2) (Fabris et al., 2007; Hofmann et al., 2010). Therefore, recovering the reduced JAG1-NOTCH2 signaling in early stage ALGS patients is an important therapeutic consideration. To improve bile duct formation and regeneration for cholestasis in ALGS, a drug treatment which increases JAG1-NOTCH2 signaling may be used in a short-term manner until bile acid regulation is reestablished.

## Investigational Therapies and Approaches

### IBAT Inhibitors

Maralixibat is a therapy being evaluated as a treatment for children with rare cholestatic liver diseases, including ALGS and progressive familial intrahepatic cholestasis (PFIC) by Mirum Pharmaceuticals. Both of these cholestatic diseases involve an interruption in the flow of bile acid from the liver, resulting in elevated bile acid levels in the liver and serum. This leads to liver disease and a variety of severe and life-altering symptoms, including stunted growth and chronic and severe pruritis. Maralixibat works by inhibiting a carrier protein called the apical sodium dependent bile acid transporter (ASBT) also known as the ileal bile acid transporter (IBAT), as shown in Figure 1. ASBT is primarily responsible for mediating the uptake of bile acids from the small intestine to the liver. Approximately 95% of bile acids are recirculated *via* the ASBT to the liver. Accordingly, a product capable of inhibiting the ASBT could lead to a reduction in bile acids returning to the liver and may represent a promising approach for treating cholestatic liver diseases. Maralixibat is currently being evaluated in the Phase 3 study in children with PFIC. In the Phase 2b clinical trial of in ALGS patients, children taking Maralixibat had statistically significant reductions in pruritus and serum bile acids compared to placebo. At the 48-week measurement, pruritus and serum bile acid reductions were maintained and improvements in skin xanthomas and quality of life were also observed. This trial has also confirmed recent findings that pharmaceutical intervention to lower serum bile acids can significantly improve transplantation-free survival rates (van Wessel et al., 2020). The Food and Drug Administration (FDA) has granted Maralixibat Breakthrough Therapy and Orphan Drug designations for pruritus associated with Alagille syndrome in patients 1 year of age and older, which will accelerate clinical development on the path to approval by the FDA (Pharma, 2020a).

Odevixibat is currently being evaluated by Albireo Pharma, Inc. as a therapy for children with cholestatic liver disease and pruritus. In Phase 2 study in children with cholestatic liver disease and pruritus, odevixibat showed reductions in serum bile acids and pruritus in most patients and exhibited an overall tolerability profile. Albireo Pharma is developing odevixibat to treat patients with PFIC, biliary atresia and ALGS. Their Phase 3 trial for PFIC recently met its primary endpoints reducing serum bile acids and pruritis. FDA and EMA also have granted an Orphan Drug Designation to odevixibat for the treatment of biliary atresia, Alagille syndrome, PFIC and primary biliary cholangitis (Pharma, 2020b).

The aforementioned investigational drugs have advanced to late-stage clinical development with promising results and are likely to constitute an important treatment option for ALGS. Their mechanism of action however, is restricted to blocking bile acid reabsorption in the intestine. A therapy addressing the root cause of the disease, namely reduced JAG1-NOTCH2 signaling, is still required to improve overall therapeutic outcomes in ALGS. The following sections will cover several technologies that have been used to address other rare genetic diseases and could be applied to developing novel treatments for ALGS (Table 1).

**TABLE 1:** Current treatments and potential future therapeutics for ALGS.

Current therapeutic modalities	
Surgical (available)	
	- Liver Transplant
	- Partial External Biliary Diversion (PEBD)
IBAT Inhibitors (in clinical trials)	
	- Maralixibat
	- Odevixibat
Potential Therapeutic Modalities	
RNA-Targeting	
	- Anti-sense Oligonucleotides (ASO)
	- Splice-switching small-molecules
	- CasRx Transcript Editing
Adenoviral Vector (AAV)-mediated gene therapy	
	- mRNA supplementation
	- CRISPR/Cas Gene Editing
	- Nuclease-based editing (ZFN’s)

### RNA Targeted Therapies

Recently, several novel therapeutic modalities have been approved for rare diseases by the Food and Drug Administration. Among these therapies are eteplirsen and nusinersen for the treatment of Duchenne Muscular Dystrophy (DMD) and Spinal Muscular Atrophy (SMA), respectively (Finkel et al., 2016; Syed, 2016). Both drugs are anti-sense oligonucleotides (ASO) which modulate splicing of their disease-causing target transcripts: dystrophin for DMD and SMN2 for SMA. Specifically, eteplirsen targets exon 51 within dystrophin transcripts, allowing for restoration of an almost full-length transcript by skipping frameshift causing mutations. Although it is estimated that only 14% of DMD patients will benefit from skipping this particular exon, therapies targeting exon-specific mutations represent a bold new therapeutic strategy for the treatment of rare genetic diseases currently lacking effective treatment (Kole and Krieg, 2015).

The application of RNA targeted therapy has notably progressed in n-of-1 clinical trials, showing great promise as a new therapeutic platform to treat rare genetic diseases using personalized exon-specific ASO’s. Milasen, a tailor-made ASO for a single patient with the rare neurodegenerative Batten disease, went from proof-of-concept to intrathecal injection in the patient within a year. This Batten disease patient has a retrotransposon insertion within the CLN7 gene that disrupts a nominal splice-site between exons 6 and 7. By targeting the retrotransposon nestled between exons 6 and 7 of the CLN7 gene, the 22-nucleotide ASO was able to restore splice-site activity to exclude the deleterious insertion (Kim et al., 2019). The reason Milasen advanced so rapidly as a therapy was due in part to the fact that it shared similar backbone chemistry with nusinersen as well as having support from the regulatory agency (Woodcock and Marks, 2019).

One of the hallmarks of ASO therapeutics described above is the array of nucleoside chemistries which enhance oligonucleotide resistance to cleavage by endonucleases and enable ASO penetration into cells (Roberts et al., 2020). Both milasen and nusinersen are 2′O-methyl-phosphorothioate oligonucleotides (2′-OMePS), while eteplirsen is instead a phosphorodiamidate morpholino oligomer (PMO). Although there are many chemistries used to balance efficacy and pharmacokinetic parameters, the aforementioned ASO’s are proof that shared nucleoside chemistry is a possibility for new therapeutics, indicating a pathway for ALGS RNA therapy development (Khvorova and Watts, 2017; Kim et al., 2019). Similar backbone structures from these approved drugs could be used as a template for the creation of ASOs which modulate the splicing of JAG1 or NOTCH2 for specific mutations to restore the reduced JAG1-NOTCH signaling in ALGS. Apart from ASOs, small molecules have yielded success in targeting mRNA splicing. One example approved by the FDA is risdiplam for SMA in August 2020. Similar to nusinersen, this drug prevents exclusion of exon 7 from SMN2 yielding a fully functional protein through the codon readthrough mechanism across a premature stop codon (Ratni et al., 2018).

Adverse events for ASO therapies consist of a wide range of categories. For example, inotersen which is used to treat hereditary transthyretin-mediated (hATTR) amyloidosis carries a label warning for glomerulonephritis, thrombocytopenia and stroke (TEGSEDI, 2019). More common side effects of ASO’s as observed in nusinersen include injection site pain, respiratory congestion, headache, nausea and fever (SPINRAZA, 2020). Major side effects observed in ASO therapy also include renal toxicity and thrombocytopenia (Chi et al., 2017; Zaslavsky et al., 2021). Current research on expanding the types of chemical modifications to ASO backbones to reduce these side effects is ongoing (Echevarría et al., 2019; Roberts et al., 2020).

Alternative technologies include mRNA editing and mRNA replacement. Recently, CRISPR technology has made it possible to edit mRNA transcripts using a Cas13 system known as CasRx (Konermann et al., 2018). This system utilizes adenoviral vectors to deliver targeted Cas13 to relevant cell types. CasRx has been used to both silence and direct alternative splicing of targeted RNA transcripts (Konermann et al., 2018; Zhou et al., 2020). It may be possible to use this approach to correct many types of mutations within JAG1 and NOTCH2 transcripts in relevant patient tissues. Although this therapy requires additional layers of development such as adeno-associated viral (AAV) delivery, further discussed below, this approach is applicable to the highly specific nature of mutations for each patient as a personalized treatment.

In addition to editing nascent transcripts in the cell, additional transcripts can be delivered as mRNA replacement. This mode of therapy is being evaluated for several other disorders such as Cystic Fibrosis and Type 2 Diabetes. Similar to RNA editing, proper cell-type specific delivery is required. Once in the cell, the transcripts are subject to translational regulation which may aid in maintaining levels within physiological range. However, the level of JAG1 expression must be well-controlled, and constitutive expression must be avoided, as it is known that JAG1 overexpression has been linked to many cancer types (Capaccione and Pine, 2013; Dai et al., 2014). Careful delivery of JAG1 mRNA to supplement reduced Jag1 levels caused by haploinsufficiency may not be completely out of the question (Aster et al., 2017). The optimal dose would overcome haploinsufficiency while avoiding oncogenic effects. To lower nonspecific expression of JAG1, transcripts can be delivered through antibody labeling of lipid nanoparticles to specific cell types (Veiga et al., 2018). Alternatively, mRNA itself can be designed to include cell-type specific microRNA binding sites that could also lower nonspecific expression of JAG1 (Jain et al., 2018). More basic study is required to evaluate JAG1 mRNA supplementation in ALGS models, as well as other non-transformed cell types to determine the optimal therapeutic window for this approach.

### Gene Therapy Using Adeno-Associated Virus

Adeno-Associated Virus (AAV) was first discovered and characterized in the 1960s and 70s and its ability to efficiently deliver genes *in vivo* made it a promising method for human gene therapy (Kaplitt et al., 1994; Carter, 2004). Wild-type AAVs contain a single-stranded ∼4.7 kb genome containing the rep gene which encodes four proteins necessary for viral replication and the cap gene that codes for the three capsid proteins (Drouin and Agbandje-McKenna, 2013). The genome of AAVs are flanked by two T-shaped inverted terminal repeats (ITRs) which are responsible for assembly and serve as origins of replication. For gene therapy, recombinant AAV (rAAV) are produced in which viral genes are replaced with the therapeutic gene of interest. The leftover ITRs guide intra-molecular assembly into circularized episomes which allows for the long-term transcription of the inserted gene (Schultz and Chamberlain, 2008). Since rAAVs are devoid of viral replication genes, they do not actively integrate into the host genome and thus have a low cytotoxicity and carcinogenesis in contrast to other viral vectors. In addition, rAAVs capsid protein can be designed to target specific cells, with over a dozen well-characterized serotypes that have already paved the way in a variety of tissues (Wu et al., 2006). These advantages have led to the broad application of rAAVs in the treatment of rare genetic disorders of which there are currently 200 ongoing clinical trials (Goswami et al., 2019).

To date two successful rAAV-based drugs have gained approval from the FDA to treat inherited genetic diseases. The first is Luxturna, which treats retinal dystrophy due to Leber Congenital Amaurosis RPE65 deficiency, a severe form of incurable childhood blindness (Cideciyan et al., 2008). The second drug is Zolgensma, which was approved to treat spinal muscular atrophy type 1, characterized by mutations to SMN1. In addition to demonstrating the safety and long-term efficacy of rAAVs for these specific diseases, the development of these two drugs also presented protocols for the large-scale production and evaluation of rAAVs that can be applied to other genetic diseases such as ALGS. Both drugs earned approval because they showed long-term improvement in their respective disease phenotypes without significant side effects (Russell, 2007; Verdera et al., 2020).

Although the virus is nonpathogenic, a significant amount of preexisting immunity is present against wild-type and recombinant capsid protein, potentially affecting the efficacy of this method across patients (Verdera et al., 2020). Some studies have also linked rAAV treatment with carcinogenesis due to insertional mutagenesis, in particular at higher doses (Russell, 2007; Nault et al., 2015). In addition to these universal limitations, the future use of rAAVs to treat ALGS is dependent on the resolution of two major challenges specific to this disease. rAAVs typically cannot deliver genes that are larger than ∼5.0 kb and the major genes involved in ALGS pathology are outside this range; JAG1 is slightly larger at 5.5 kb and NOTCH2 is 7.4 kb. One workaround would be to design a shortened, but functional, isoform of JAG1 or NOTCH2. Such a microgene approach has shown promise in preclinical models of Duchenne muscular dystrophy, which involves mutations in the 11.5 kb dystrophin gene, but has yet to achieve clinical proof-of-concept in patients (Duan, 2018). Alternatively, either of the genes can be split between two co-administered rAAV vectors with the reconstitution of the full gene facilitated by ITRs that can be removed during endogenous splicing events (Nakai et al., 2000). The second challenge is the oncogenic nature of Notch signaling components which inherently requires a more targeted approach, as previously mentioned (Xiu et al., 2020). Currently, many rAAV serotypes can efficiently transduce hepatocytes and have been used as delivery vectors for other liver diseases such as Crigler-Najjar. Safe and specific JAG1 expression may require vectors which infect a multitude of cell types in the liver as well as the introduction of regulatory elements that confer tissue- and cell type-specific expression (Manno et al., 2006; Nathwani et al., 2014; Collaud et al., 2019). Cell type specificity may be of particular importance since the intracellular domain of Jag1 (JICD) has been reported to prevent NICD dependent transcriptional regulation (Kim et al., 2011).

### Nuclease-Based Genome Editing

Permanent alteration of the genome *in vivo* is accomplished using four main types of nucleases: zinc-finger nucleases (ZFNs), transcription activator-like effector nucleases (TALENs), meganucleases and CRISPR/Cas-based nucleases (Silva et al., 2011; Gaj et al., 2013). These nucleases induce double stranded breaks within a specific region of DNA, which then allows for the tandem incorporation of an exogenously introduced gene at that cleavage site through a process called homology-directed repair (HDR) (Silva et al., 2011; Gaj et al., 2013). While ZFNs, TALENs and meganucleases contain DNA binding domains that can be engineered to bind to specific regions of DNA, CRISPR/Cas-based nucleases are unique in that they are guided by an RNA oligomer that is specific to a particular DNA sequence (Gaj et al., 2013). Practical delivery of these nucleases and the oligo targeting the gene of interest is usually mediated by viral vectors, such as rAAVs, though non-viral methods also exist (Wang et al., 2017b). The advent of CRISPR has greatly accelerated our ability to perturb the expression of most genes due to the simplicity of its targeting mechanism. The major advantage of using this technology to treat ALGS would be the permanence of the therapy relative to other techniques, since the mutation in the genomic DNA is corrected and would persist through cell division.

Though there are currently no FDA-approved *in vivo* gene editing therapeutics, many promising candidates are undergoing clinical trials (Li et al., 2020). ZFNs have only seen moderate success in the generation of genome edited cells *ex vivo*, while TALENs have only shown efficacy in animal models (Li et al., 2020). EDIT-101 made history in 2018 as the first ever CRISPR-Cas9-based genetic therapy to enter Phase I/II clinical trials. This therapy seeks to correct a point mutation in intron 26 of the CEP290 gene which introduces an alternative splice site resulting in a premature stop codon and is the most common mutation underlying Leber Congenital Amaurosis (Maeder et al., 2019). In this treatment, AAV5 delivers the coding sequence for the Cas9 protein along with two guide RNAs. These guide RNAs flank the sequence around the mutation in intron, IVS26, and lead to its deletion or inversion, abolishing the splice site. This method can be applied to the mutations present in JAG1 or NOTCH2 in a similar fashion retaining the ability to package an entire treatment payload in just one rAAV. Other methods, like ZFNs, require up to three separate vectors to deliver the coding sequences for the nucleases and the correct transcript. This has limited the efficacy of using ZFNs, as was the case with SB-915 which failed in Phase I/II clinical trials since the uptake of all three of its vectors in a single cell was needed for any successful gene editing to occur (Sheridan, 2018). Recently, the CRISPR toolbox has expanded further to allow for the editing of single base mutations directly by coupling an inactive Cas9 nuclease with cytidine deaminase (Gaudelli et al., 2017). While this method has not been applied in the clinic yet, it has been validated in murine models showing the ability to permanently correct genes in a broad variety of tissues, including the liver (Levy et al., 2020).

Nuclease-based treatments often fail in the clinic because of off-target effects, the low rate of gene integration by HDR and host immune response (Li et al., 2020). Continued development has addressed some of these limitations, particularly for Cas-based nucleases, improving integration efficiency while limiting the induction of double stranded breaks in other regions of the genome (Xie et al., 2014; Song et al., 2016). Since the CRISPR/Cas system is derived from a bacterial source however, overcoming the high innate immunity present in human populations to these nucleases is likely to continue to impact efficacy (Charlesworth et al., 2019). Gene editing could aim to correct single mutations or replace larger segments covering multiple mutations found in patients. Theoretically, gene editing could also aim to correct the entire mutant copy of the JAG1 gene already present in patients with Alagille Syndrome. While ZFNs, TALENs and meganucleases can be designed to target any sequence for cleavage, Cas nucleases require the presence of a PAM sequence near the targeting site, potentially limiting what parts of the JAG1 gene can be corrected. The emergence of Cas-based point mutation editing is particularly promising for ALGS as it potentially alleviates the largest limitations of nuclease-based gene editing, offering further options for the development of gene editing-based therapeutics.

## Perspectives for ALGS Therapeutics Development

Recent advances in organoid technology and patient-derived induced pluripotent stem cells (iPSC) have brought new modeling systems to the hunt for therapies of rare diseases. For Alagille Syndrome in particular, the difficulty of identifying therapeutics which modulate the Notch signaling pathway with a desired functional outcome would require such technologies. Improvements in iPSC liver cell differentiation have allowed modeling of human liver diseases with patient-derived cells. Human iPSCs can be generated from patient cell samples such as peripheral blood, dermal fibroblasts, urine, hair follicles and keratinocytes (Takahashi et al., 2007; Farkhondeh et al., 2019). The use of iPSC-derived hepatocytes, cholangiocytes and stellate cells will assist in advancing compound screening as well as drug efficacy evaluation. Furthermore, patient-derived iPSCs carry the unique genetic background of the donor which allows to precisely model disease-specific pathophysiology and phenotypes observed in complex genetic disorders such as ALGS. Liver cells derived from iPSCs can be produced in a large quantity and would be a new direction for the broader context of liver disease drug discovery.

Patient-derived organoids are a newly established platform for disease modeling and development of precision medicine (van de Wetering et al., 2015). Organoid models generated from mutation-specific iPSC lines can be used to establish disease-relevant phenotypes to evaluate and predict clinical efficacies for lead compounds. ALGS patient-derived iPSCs and their differentiated organoids represent a more relevant disease model system as they share the same genetic background as the patients they come from. Furthermore, these liver organoids can share similar disease phenotypes as observed in patients, providing further biological context for drug development. Intestinal organoids for example, have been used as a model for evaluating cystic fibrosis variants for drug development (Chen et al., 2019). Single-cell RNA-sequencing of organoids can yield additional valuable information about the transcriptional landscape of interacting cells. Using a combination of scRNA-Seq and immunostaining it may be possible to identify new targets for drug development. By having a patient-specific context for disease, as with ALGS, these technologies can be leveraged to enable the discovery of novel small-molecule and RNA therapies (Akbari et al., 2019; Sakabe et al., 2020). Although compound screening has expanded into the realm of ultra high-throughput technologies, i. e 1536-well screening of >1 million molecule libraries, incorporation of high-content imaging and machine learning for image analysis and modeling will accelerate drug development. Phenotypic screening in this context has already been developed for several orphan diseases like Batten’s disease and Niemann-Pick Type C (NPC) disease (Chandrachud et al., 2015; Sun et al., 2017; Pugach et al., 2018).

**FIGURE 3:**
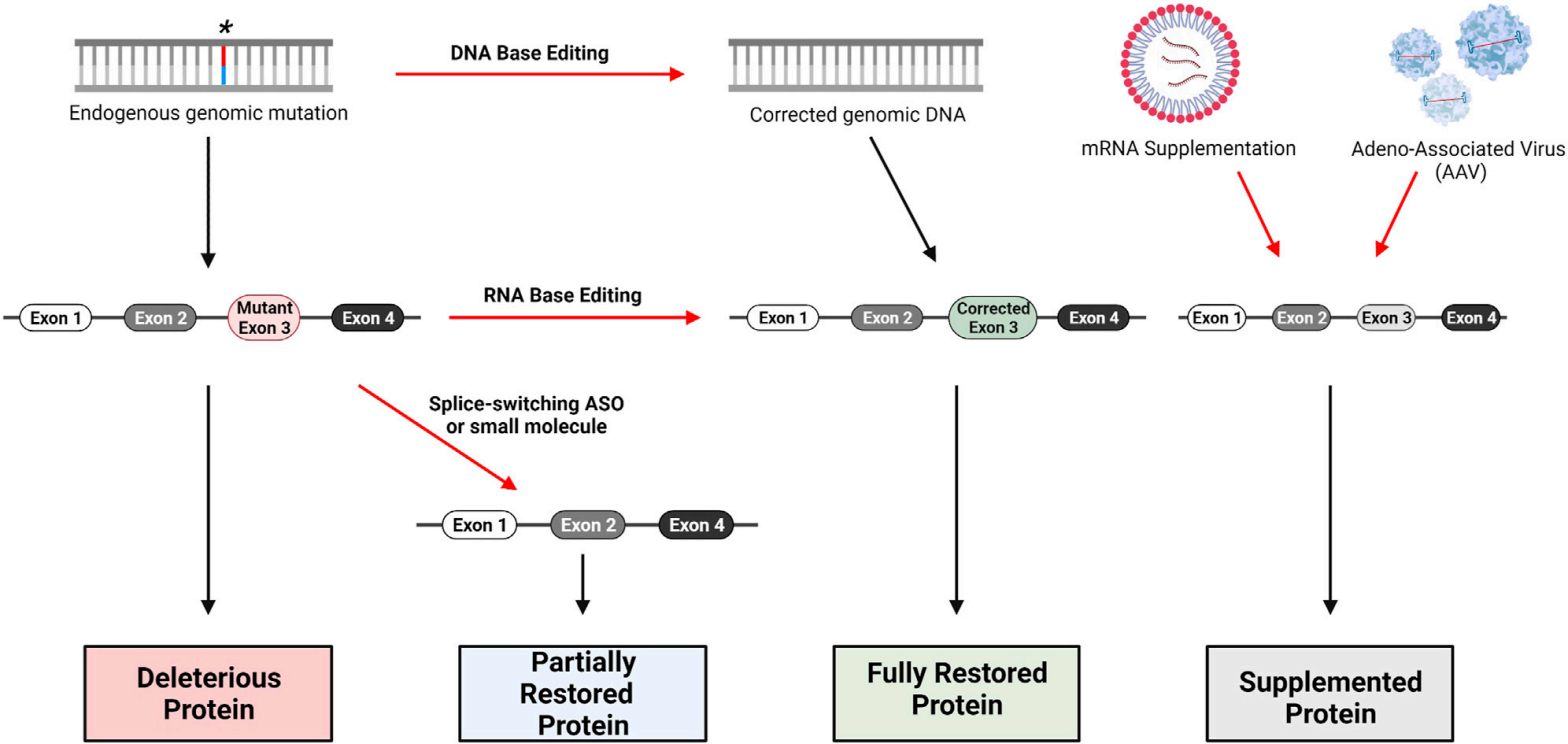
Gene therapy approaches for loss-of-function genetic disorders. DNA base editing technologies include CRISPR/Cas9, Zinc-Finger Nucleases (ZFN) and Transcription Activator-Like Effector Nucleases (TALENS). RNA base editing of pre-mRNA can be performed with the CasRx system, utilizing Cas13. Skipping of mutant-containing exons during mRNA processing can be performed with Antisense Oligonucleotides (ASO) specifically designed to bind relevant intron-exon junctions; small-molecules such as risdiplam are also capable of altering splicing. Traditional replacement therapy including mRNA supplementation and AAV delivery are possible as well, though several factors complicate this approach for Alagille Syndrome as we have discussed.

The technologies presented here have the potential for development of therapeutics to treat all types of mutations in JAG1 or NOTCH2 that have been identified as pathologic variants for ALGS (Figure 3). The design of truly personalized RNA therapies as with Milasen is conceivable for ALGS. However, given the publicly available data surrounding ALGS mutations in both JAG1 and NOTCH2, it should be possible to generate RNA or gene editing therapies for subpopulations of patients that carry mutations in the same region of either of these genes. In addition to targeting specific mutations represented in the JAG1 and NOTCH2 mutational database, it may also be possible to develop long-term or even permanent treatments that can be applied to all patients using rAAVs or nuclease-based gene editing. The development of a targeted ALGS therapy would be a much-needed improvement over current approaches which only ameliorate symptoms of disease.
